# The exploration of network motifs as potential drug targets from post-translational regulatory networks

**DOI:** 10.1038/srep20558

**Published:** 2016-02-08

**Authors:** Xiao-Dong Zhang, Jiangning Song, Peer Bork, Xing-Ming Zhao

**Affiliations:** 1 Department of Computer Science and Technology, School of Electronics and Information Engineering, Tongji University, Shanghai 201804, China; 2 Shanghai Water (Ocean) Administrative Service Center, Shanghai 200050, China; 3 Tianjin Institute of Industrial Biotechnology, Chinese Academy of Sciences, Tianjin 300308, China; 4 Department of Biochemistry and Molecular Biology, Faculty of Medicine, Monash University, Melbourne, VIC 3800, Australia; 5 European Molecular Biology Laboratory, Meyerhofstraße 1, Heidelberg 69117, Germany

## Abstract

Phosphorylation and proteolysis are among the most common post-translational modifications (PTMs), and play critical roles in various biological processes. More recent discoveries imply that the crosstalks between these two PTMs are involved in many diseases. In this work, we construct a post-translational regulatory network (PTRN) consists of phosphorylation and proteolysis processes, which enables us to investigate the regulatory interplays between these two PTMs. With the PTRN, we identify some functional network motifs that are significantly enriched with drug targets, some of which are further found to contain multiple proteins targeted by combinatorial drugs. These findings imply that the network motifs may be used to predict targets when designing new drugs. Inspired by this, we propose a novel computational approach called NetTar for predicting drug targets using the identified network motifs. Benchmarking results on real data indicate that our approach can be used for accurate prediction of novel proteins targeted by known drugs.

Protein post-translational modifications (PTMs) play crucial roles in regulating the activity, localization and interactions of proteins in distinct cellular processes, such as signaling cascades and cellular differentiation^1^. Among various types of PTMs, phosphorylation is among the most common ones and has been studied extensively. Via phosphorylation, a kinase switches on the activity of a protein by adding a phosphate group to its residue(s), thereby regulating its activity and function. Phosphorylation is involved in numerous cellular processes, e.g. cell cycle and signal transduction. Proteolysis is another common type of PTM, which is an irreversible process that involves degradation of a target protein via the hydrolysis of a peptide bond, where cleavage of the peptide bonds by the protease leads to decomposition of the substrate. Proteolysis has a critical role in apoptosis and immune response^2^. Both types of the above enzymes, i.e. kinases and proteases, have been used as effective drug targets in the treatment of cancers.

Recently, extensive functional crosstalks between kinases and proteases have been observed in cell proliferation, apoptosis, and metastasis, which make it an attractive topic to develop new agents for treating cancers by targeting the crosstalks between kinases and proteases^3^. Indeed, effective combinatorial anticancer therapies that target the crosstalks between kinases and proteases have already been proposed. For example, Zhou *et al.* found that inhibiting ADAM would affect HER3 and EGFR pathways in non-small cell lung cancer (NSCLC), and offered a new promising therapy option^4^. Lu *et al.* indicated that targeting the two proteases MMP1 and ADAMTS1 as well as EGFR signaling in bone stroma could be a promising therapeutic approach for treating bone metastasis in breast cancer^5^. Therefore, exploring the crosstalks between kinases and proteases as well as their regulated PTMs could provide important insights into the underlying mechanisms of diseases and facilitate the development of novel effective therapies.

Since complex biological systems consist of distinct kinds of molecules that interact with each other, it is reasonable to represent a biological system as biological networks, e.g. signaling networks and protein-protein interaction networks^5^. Recently, it is found that biological networks are generally composed of small functional blocks, i.e. network motifs, that appear with higher frequencies than expected^6^. These small network motifs consist of limited number of nodes, but are important for the functionality and robustness of biological networks. For example, some motifs are found to be crucial to achieve biochemical adaptation. Therefore, it is not surprising that some motifs are significantly conserved from bacteria and yeast to human^7^. In literature, some network motif detection tools have been developed, such as MFinder^8^, FANMOD^9^, Grochow-Kellis^10^, Kavosh^11^ and G-Tries^12^, and the strength and weakness of distinct approaches have been explored^13^.

In this study, we assembled a post-translational regulatory network (PTRN) that comprises kinases/phosphatases and proteases as well as their respective substrates, with which we elucidated the crosstalks between phosphorylation and proteolysis. In particular, we identified significant network motifs composed of the regulatory interplays between the two PTMs. By investigating these motifs, we found that they were significantly enriched with drug targets, suggesting the possibility of exploring these conserved motifs as potential drug targets. Inspired by this, we developed a novel approach for predicting drug target proteins by considering the topology and conservation of the network motifs. Benchmarking results on real data demonstrate the competitive performance of our proposed approach compared with existing popular methods, indicating that the network motifs are indeed effective for predicting drug targets. Furthermore, we predicted some novel targets for known drugs, which were validated by drug target information from another database, implying the predictive power of our approach. In addition, we found that the regulatory network motifs can help design multi-component or combinatorial drugs, where interventions targeting multiple proteins within a motif may improve therapeutic effects.

## Results

### Identification of network motifs in PTRN

**Figure 1:**
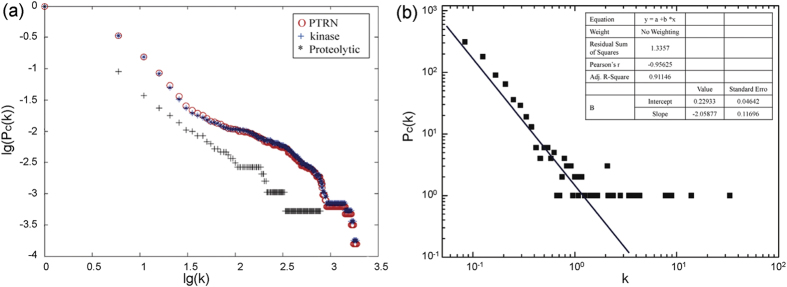
The degree cumulative distribution of PTRN, Kinome and Proteolytic networks, where *k* is the degree and *P*_*C*_*(k)* is the percentage of nodes with the degree no less than *k*. (**a**) The degree cumulative distribution of the three networks. (**b**) The fitting of the power-law distribution for the Proteolytic network.

We obtained a PTRN composed of 33,930 regulations among 6,412 proteins, including 375 kinases/phosphatases and 205 proteases. In the PTRN, the nodes in the PTRN are either enzymes or their substrates. A directed link from an enzyme to its substrate will be laid if this relationship has been reported in literature. In this way, most of the links are unidirectional edges from kinases or proteases to their substrate proteins. If a pair of enzymes (either kinase or protease) were reported to be regulated with each other in public databases, the edge between them will be denoted as a bidirectional link. Since the biological networks have been reported to be scale-free networks, we investigated the topological structures of the PTRN as well as its Kinome (kinase-substrates) and Proteolytic (protease-substrates) networks. Figure 1a shows the degree cumulative distribution of the three networks, from which we can see that only the Proteolytic network follows the power-law distribution, and the others follow the right-skewed distribution. Figure 1b shows the fitting of the power-law distribution for the Proteolytic network as well as corresponding parameters.

**Figure 2:**
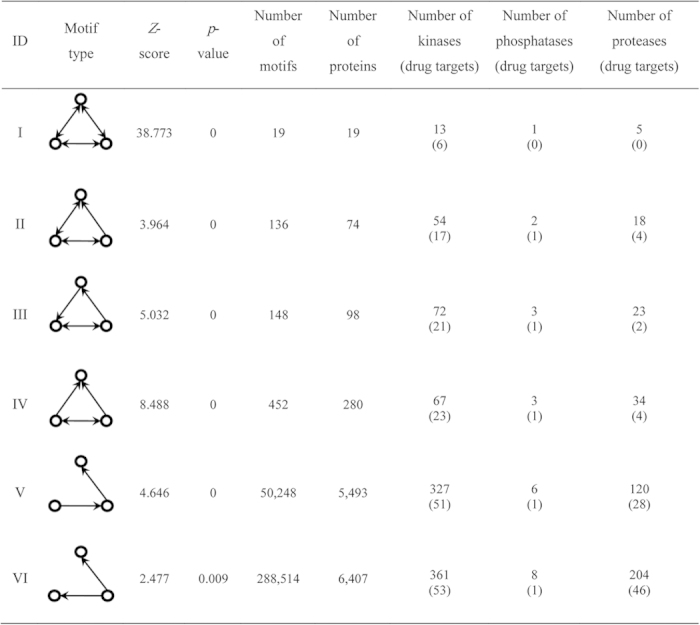
Six significant network motifs identified from the PTRN using the FANMOD tool.

The FANMOD tool^9^ was utilized here to identify network motifs due to its efficiency and convenience. Here, we only detected the three-nodes motifs and larger ones were not considered due to the high computational costs of detecting larger motifs consist of more nodes. In particular, we focused on the motifs that comprised at least one kinase/phosphatase and one protease to explore the crosstalks between kinases/phosphatases and proteases. As a result, we identified six significant motifs that occurred with higher frequencies than expected (Supplementary Tables S1,S2,S3,S4,S5,S6). Figure 2 provides the details of the six motifs we identified, including the number of enzymes involved and the significance scores of the motifs. They were classified into two groups: with feedback loops, i.e. motifs I, II and III; or without feedback loops, i.e. motifs IV, V and VI. Among these motifs, motif VI with a single-input like structure^14^ was the most common with the highest frequency, while motifs I–IV had co-regulated enzymes.

### Enrichment of drug targets in the PTRN motifs

By focusing on the six motifs shown in Fig. 2, we want to see whether these motifs tend to contain drug targets, i.e. whether drug target proteins are enriched in the motifs. We investigated the targets of drugs from different therapeutic categories, and found that the six motifs were significantly enriched with proteins targeted by drugs with specific effects as shown in Table 1. Using the first level of the Anatomical Therapeutic Chemical (ATC) classification system, we noted that all six motifs contained proteins targeted by antineoplastic and immunomodulating agents (with the ATC code L). Table 1 summarizes the therapeutic categories whose targets were significantly enriched in the motifs based on the Fisher’s exact test^15^ with Holm correction considering the possibility of multiple therapeutic effects associated with one drug. In particular, motifs II–IV and VI were found to be enriched with proteins targeted by alimentary tract and metabolism agents (with the ATC code A), motif IV was enriched with target proteins of blood and blood-forming organ agents (with the ATC code B), while motif V was targeted by various agents, including those used to treat disorders of the respiratory (with the ATC code R), cardiovascular (with the ATC code C), neoplastic (with the ATC code N), dermatological (with the ATC code D), and nervous systems (with the ATC code N).

**Figure 3:**
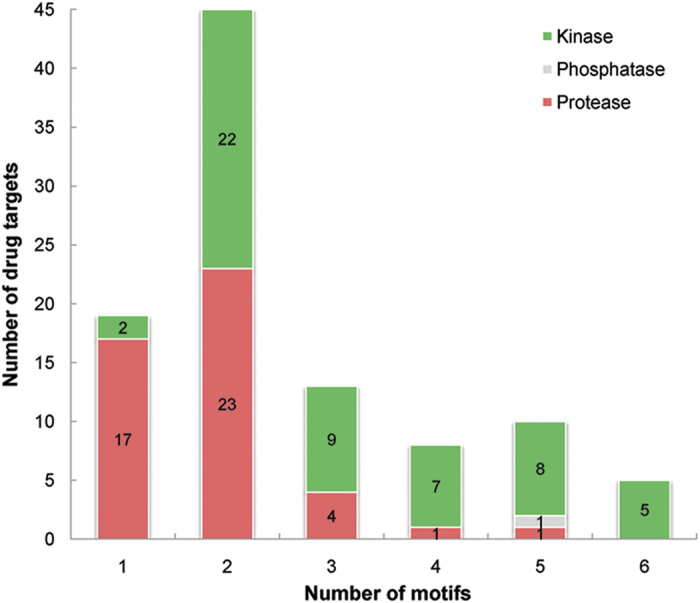
The distribution of kinases/phosphatases and proteases acting as drug targets across the six motifs.

Since the enzymes were widely used as drug targets, we further investigated the drug targets contained in the above six motifs. Figure 3 shows the distribution of drug targets across the six motifs, from which we can see that the drug target proteins are uniformly distributed across the motifs, and only very few drug targets occur in more than 3 motifs. The details can be found in Supplementary Table S7. In other words, the enrichment of drug targets in network motifs is not due to the dominance of certain drug targets. For example, the five proteins SRC, AKT1, FYN, MAPK1 and MAPK3 appeared in all six motifs, while 19 enzymes, including PCSK1, MMP17 and PIM1, participated only in one of the six motifs.

**Figure 4:**
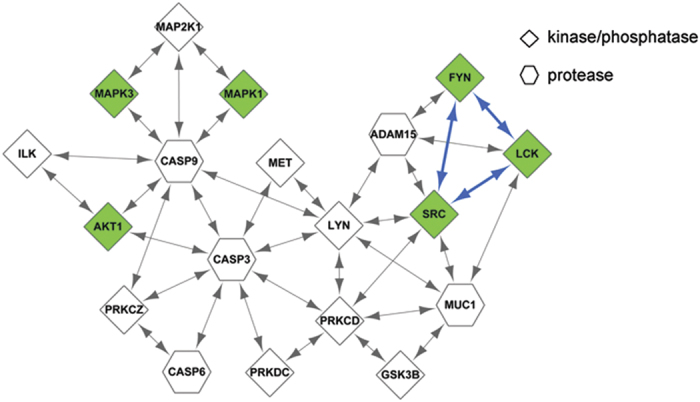
A network consists of proteins as well as their interactions that occur in motif I. Green nodes denote drug targets and blue edges denote the interactions between the drug target proteins.

The enrichment of drug targets in the motifs we identified indicates that the regulations between kinases/phosphatases and proteases might play important roles in disease treatment. Figure 4 shows the network of consists of proteins as well as their interactions that occur in motif I, which is actually a subnetwork of PTRN, where there exist extensive crosstalks between kinases and proteases. For example, three drug targets, i.e. MAPK1, MAPK3 and AKT1, regulate the protease CASP9, thereby suggesting the important role of this protease. Due to the inhibition of MAPK1 or MAPK3, CASP9 cannot be phosphorylated, which leads to the activation of CASP3 and its downstream caspases so that the cellular destruction is initiated^16^. In addition, the inhibition of AKT1 leads to the dysregulation of alternative splicing of CASP9, thereby providing an efficient method for treating NSCLC^17^. Similarly, the drug targets FYN, LCK and SRC regulate the protease ADAM15. It has been found that the inhibition of the interaction between ADAM15B and SRC could be used as an effective therapy to treat breast cancer^18^. Both FYN and LCK belong to the SRC family, thus it is expected that inhibition of the interaction between each of the two kinases and ADAM15 could obtain similar effects^19^. Based on the PTRN map shown in Fig. 4, we can see that although proteases are not targeted directly by drugs, they may play important roles in the treatment of diseases due to the presence of the regulatory interplay between the kinases targeted by drugs and the proteases. Given that motif I contains proteins that are targeted significantly by anti-neoplastic agents, we expected that targeting the specific crosstalks between proteases and kinases within this motif might help to improve the therapeutic efficacy of cancer treatment.

### Network motifs as targets of combinatorial drugs or multi-target agents

**Figure 5:**
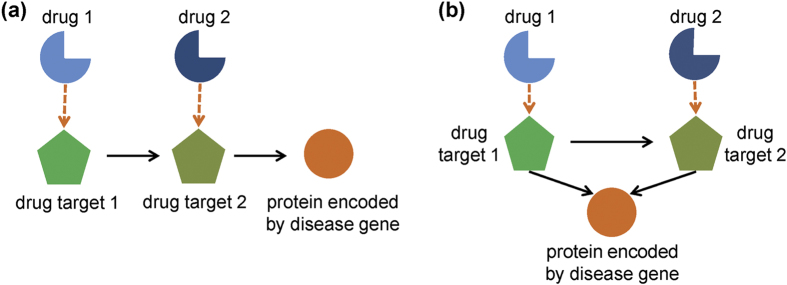
Regulation of proteins encoded by disease genes by a pair of interacting proteins within the same motif. (**a**) Drugs act on disease gene productions via the regulation of a pair of interacting proteins in a sequential and cascade manner. (**b**) Drugs act on proteins encoded by disease genes by targeting an interacting protein pair in a parallel manner.

As shown in Fig. 5, we found that some proteins encoded by disease genes could be regulated by a pair of interacting proteins in a cascaded or parallel manner. We assumed that the drug pairs that targeted these protein pairs were more likely to have similar therapeutic effects. By investigating the therapeutic effects of the drugs that target an interacting protein pair within the same motif and subsequently calculating their therapeutic similarity with equation (2), we found that the drugs shown in Fig. 5b were more likely to share therapeutic effects than those shown in Fig. 5a. For example, for the four cases in motif I (Supplementary Table S8a), the drugs that target an interacting protein pair were exactly the same one as listed in Table 2. For motif II, the drug pairs targeting 17 cases had average therapeutic similarity score larger than 0.50, whereas each one from 12 cases was targeted by the same drug (Supplementary Table S8b). Similar results were also obtained for motif IV, where 8 cases were targeted by drugs with similar therapeutic effects (Supplementary Table S8c–f). To investigate whether this phenomenon is due to the interacting drug targets, we compared the similarities of the drugs targeting the interacting proteins in- or out-side of the network motifs. We found that the drugs target a protein pair in cascade or parallel manner within a network motif are significantly therapeutically similar than those targeting interacting proteins outside of the motif (with *p*-values of 0.0152 and 2.8908e-11, respectively), indicating that the drugs targeting the same network motifs are possibly more similar.

The above findings indicate that the drugs targeting the same motif tend to have similar effects, thereby suggesting that the motif might be used as a potential drug target, especially when considering the development of novel multi-target therapies. For example, dasatinib is a multi-target agent used to treat patients suffering from chronic myelogenous leukemia (CML) and Philadelphia chromosome-positive acute lymphoblastic leukemia^20^. Examining the proteins targeted by dasatinib in motif I can help to elucidate the mechanism of action of this drug. Among the target proteins, LCK and FYN are important for T-cell antigen receptor signal transduction^21^. FYN and SRC are also effectors of EGFR-mediated glioblastoma^22^ and play key roles in the growth and motility of glioblastoma. Thus, it is not surprising that dasatinib can be used to treat cancers in an efficient manner by targeting these proteins^23^. In motif V, marimastat is a synthesized matrix metallo-proteinase (MMP) inhibitor^24^ that targets motifs containing proteins MMP14 and MMP13. In motif II, marimastat targets motifs containing MMP2 and MMP9. Previous studies indicate that MMPs are responsible for the degradation of the extracellular matrix and they are related closely to tumor invasion and metastasis^25^. MMPs promote the formation of several tumors, thus marimastat has been used in the treatment of patients with cancers, including advanced pancreatic cancer and gastric cancer^26^,^27^.

In addition to the multi-target agents that regulate motifs, as described above, we tested whether drugs that targeted the same motif could be combined to improve the therapeutic efficacy. To answer this question, we extracted drug combinations from the Drug Combination Database^28^, which is an online resource that collects approved drug combinations from the US Food and Drug Administration as well as previous publications. We retained 269 drug combinations for further analysis after discarding those without valid target information, with which we investigated whether the drugs targeting our identified motifs could be used concurrently to obtain a better therapy. In motif II, the two drugs trastuzumab and gefitinib target ERBB2 and EGFR, respectively. A combination of these two drugs has been used clinically to treat breast cancer^29^. Trastuzumab down-regulates the expression of ERBB2 and prevents both cell proliferation and tumor formation^29^, while gefitinib inhibits the activity of tyrosine kinase EGFR to inhibit the progression of cell cycle and tumor formation by arresting receptor autophosphorylation and the signal transduction process^30^. Furthermore, both ERBB2 and EGFR are components of the ERBB signaling pathway, which can also affect the MAPK and PI3K-AKT signaling pathways that are related to cell proliferation and differentiation. This agrees with our previous report that drug combinations tend to target interacting and crosstalking pathways^31^,^32^. Motif V encompasses two proteins, i.e. ABL1 and the mammalian target of rapamycin (MTOR), which are targeted by imatinib and sirolimus, respectively. A combination of these two drugs was already known to be an effective anticancer therapy for CML^33^. Although CML cells were known to be resistant to the ABL inhibitor imatinib, the resistant CML cells became sensitive to imatinib when it was administered together with sirolimus that inhibits MTOR^34^. Except for the examples given above that contain two kinase drug targets or two protease drug targets in the same motif, we also found the crosstalk between a pair of kinase and protease targeted by a pair of drugs. For instance, the kinase IGF1R and protease MMP2 were involved in 650 cases of motif V. MMP2 is located in the downstream of IGF1R-induced signaling pathway, and the inhibition of IGF1R will affect the dissemination of hepatocellular carcinoma (HCC) cells^35^. IGF1R and MMP2 were targeted by drugs with different therapeutic effects (with ATC code A and C respectively). Despite the combination of drug pairs targeting these two proteins has not been reported, the functions of these two proteins imply promising perspective of combinatorial therapy for HCC. Overall, these results indicate that the motifs identified here can be used as potential targets for combinatorial therapy and they may facilitate the design of new multi-target or combinatorial drugs.

### Prediction of drug targets using network motifs

From the analysis in previous sections, we can see that the identified motifs are enriched with drug targets and some combinatorial or multi-component drugs target multiple proteins in the motifs. Therefore, we suggested to use the motifs instead of single proteins as drug targets considering the functional importance and conservation of network motifs, and presented a new computational approach called NetTar to predict drug targets. Here, we only considered agents belonging to drug categories whose targets were enriched in the six motifs, i.e. the categories with ATC codes A, B, C, D, L, N and R. For example, all the six motifs were targeted by antineoplastic and immunomodulating agents (with ATC code L). For the proteins in the PTRN, using known antineoplastic drug targets as positive set while the rest as negative set, NetTar will predict whether a new protein is targeted by an antineoplastic drug by investigating the functional similarity between the protein and those sharing the same motif structure and targeted by the antineoplastic drug from the positive set (see Methods).

Using drug targets extracted from DrugBank^36^ as the gold standard, we evaluated the predictive power of NetTar by performing leave-one-out cross-validation tests, where each target protein was selected as the test set while the rest were used as the training set. This procedure was repeated *n* −1 times, assuming that there were *n* target proteins. In particular, we predicted the target proteins of drugs associated with ATC codes A, B, C, D, L, N and R. Moreover, we compared the performance of our method with that of the popular nearest profile method^37^ using the functional similarity instead of the sequence similarity between a pair of proteins. In the latter method, one protein was regarded as the target of a drug if it was functionally similar to those in the positive set. Furthermore, we compared NetTar with the approach proposed by Zhao *et al.* based on network topology^38^, where one protein was predicted as a drug target if the protein is close to known drug target.

Table 3 shows the performance of our proposed NetTar, the nearest profile method (referred to as NNfun) and Zhao *et al.*’s From the results, it can be clearly seen that NetTar significantly outperforms Zhao *et al.*’s and NNfun across all therapeutic categories, with the single exception of ATC code C, which demonstrates the predictive power of our approach. Despite the overall performance (i.e. *F1*) of NNfun is better, NetTar gets better precision results. The excellent performance of NetTar also indicates that the network motifs can facilitate the elucidation of the mechanisms of drug actions, thus they may have great potential as effective drug targets. To verify the robustness of our NetTar, we considered two distinct phosphorylation datasets, one from Tan *et al.*^39^ (the phosphorylation network composed of 22,882 kinase-substrate regulations including 106 kinases and 5,031 substrates) and the other from PhosphoSitePlus^40^ (the phosphorylation network composed of 3,446 regulations between 305 kinases and 1,593 substrates), where the two datasets have only a small overlap of 589 regulations among 63 kinases and 468 substrates. We investigated the robustness of our NetTar on the two PTRNs constructed based on of the two phosphorylation datasets and the proteolysis dataset used in our work, and the performance of NetTar on these two datasets can tell its robustness to possible false positives and false negatives. Note that some of the six motifs may be not significant anymore in the two new networks and will not used for drug target prediction. For a fair comparison, we applied NNfun to the two networks to predict drug targets. The good performance of NetTar on distinct datasets shown in Table 3 indicates the robustness of our approach against possible false positives and false negatives.

### Identification of novel drug targets

After demonstrating the effectiveness of the NetTar method, we also explored the possibility of predicting novel drug target proteins using the network motifs we identified. Given drugs labeled with ATC codes A, B, C, D, L, N and R, we tried to predict their novel target proteins. Our criterion was that given a new protein located in any of the six motifs, it was predicted to be targeted by the agents whose target proteins share the same topological structure with the protein in corresponding motifs and have similar functions.

To validate our predictions based on the drug target information from Drugbank, we used the drug targets from the Therapeutic Target Database (TTD)^41^ and Search Tool for Interactions of Chemicals (STITCH)^42^. Among our 4900 novel predictions (Supplementary Table S9), 205 proteins were validated to be targeted by drugs in TTD and STITCH (see Table 4). For example, CASP9 was predicted to be a target of sorafenib by NetTar, where the compound was used for the treatment of unresectable hepatocellular carcinoma and advanced renal cell carcinoma and the other two protein, MAPK1 and RAF1, from motif II has been found to be related to the diseases^16^,^43^,^44^. The drug-protein interaction was also validated in STITCH. Furthermore, the drugs targeting MAPK1 and RAF1 were all annotated with ATC code L and have therapeutic similarity of 0.75, thereby indicating that CASP9 might also be potential targets of these drugs given its important role in programmed cell death^45^. In addition, NFKB2 was identified as a target of alimentary tract and metabolisma agents by NetTar due to its high functional similarity with the known target protein IKBKB. In particular, NFKB2 was predicted to be a target of sulfasalizine used for the treatment of rheumatoid arthritis and was validated in TTD^46^. In summary, the validation of our predicted targets for known drugs in public databases implies the predictive power of the NetTar approach.

Although some predictions could not be verified in public databases, they are not necessarily false positives. For example, PTH2R was predicted as the target of drugs annotated with ATC code D, and the protein has been found to be associated with psoriasis and psoriatic disorders in TTD. MAP2K7 was identified by NetTar as a target of antineoplastic agents while the protein has been reported to be associated with prostate cancer in TTD. Despite some drugs cannot be verified directly, the drugs involved in some predictions may have similar therapeutic effects as those targeting the proteins in the predictions. For instance, the protein MAP2K1 was predicted to be the target of antineoplastic and immunomodulating agents, which was not reported in DrugBank. It has been found that MAP2K1 could be targeted by the inhibitor U0126 that was used in the treatment of medulloblastoma metastasis^47^, indicating the potential of the protein to be antineoplastic drug target. Overall, the competitive performance of our NetTar method suggests that our identified network motifs could facilitate the prediction of drug targets, or the motifs themselves could be explored as targets to develop multi-target or combinatorial therapy in translational applications. Our results also demonstrate the complementary benefits of our proposed method with other approaches, e.g. the near profile method, and it is possible that improved methods could be developed in future studies to enhance the performance when predicting novel drug targets by combining different but complementary methods.

## Discussion

Phosphorylation and proteolysis are the two most important types of PTMs in biological systems, where their crosstalk has been implicated in numerous pathological processes and diseases. In this study, we constructed a PTRN that encompassed kinases/phosphatases and proteases as well as their corresponding substrates to investigate functional crosstalks between the two PTM processes. In particular, we identified significant network motifs involving the regulatory interplay between kinases/phosphatases and proteases. We identified six such network motifs and found that they were significantly enriched with known drug target proteins, suggesting the potential of network motifs as useful drug targets in subsequent translational studies. Despite the controversy over the definition of network motifs as well as their relatedness to biological functions^14^, the network motifs detected here are indeed enriched with drug targets and can serve as potential targets.

Moreover, the network motifs identified here provide useful insights into the underlying mechanisms of drug actions that target the motifs. For example, some disease genes were regulated by a pair of interacting proteins from the motifs and the drug pair targeting such protein pair were found to have similar therapeutic effects. This suggests that there may be functional redundancy between pairs of interacting proteins as described in our previous work^48^ and drugs that target both proteins may obtain a better therapeutic effect. This observation has been confirmed by the clinical use of multi-target drugs such as dasatinib. Furthermore, the network motifs provide alternative useful routes for combinatorial therapy. We found that drugs that target proteins within the same motif may be administered concurrently. For example, trastuzumab and gefitinib respectively target ERBB2 and EGFR from the same motif, and they have been used clinically in combination to treat breast cancer. Another pair of drugs, imatinib and sirolimus, targeting ABL1 and MTOR has been used in combination to treat CML. It should be noted that these conclusions are consistent with our previous findings that effective drug combinations can be obtained based on combinations of their target proteins^49^. These findings suggest that functional network motifs instead of single proteins should be considered as targets when designing new drugs in the future.

Given that network motifs are generally functionally conserved and that the characteristic network motifs we identified are significantly enriched with drug targets, we assumed that proteins within the same motif are more likely to be targeted by drugs with similar therapeutic effects. Therefore, we developed the novel NetTar approach to predict potential drug targets based on the identified network motifs. Benchmarking results on real data demonstrated that this approach outperformed the popular nearest profile approach. Despite we only compared our approach with the nearest profile approach, the good performance of our NetTar approach makes it clear that the network motifs indeed can help identify novel targets for known drugs, and are therefore well complementary to existing approaches. The verification of our novel predictions in public databases also indicates the predictive power of network motifs for identifying novel drug targets.

In this paper, we only considered the three-nodes motifs without considering larger motifs due to the high computational cost. Generally, the first two steps in network motif detection are sampling subgraphs and generating random networks. The complexity of sampling subgraphs of *n* nodes in a network is *O(N*_*s*_*K*^n−1^*n*^n+1^), where *K* is the average node degree in the network and *N*_*s*_ is the number of subgraphs sampled. The complexity of generating a random network is *O(T*_*s*_*N*_*e*_), where *T*_*s*_ is the switch times per edge and *N*_*e*_ is the number of edges of the real network. The overall complexity of these two steps is *O(N*_*s*_*K*^n−1^*n*^n+1^*(1* + *N*_*r*_) + *N*_*r*_*T*_*s*_*N*_*e*_), where *N*_*r*_ is the number of random networks^50^. It can be seen that with the size of motif grows, the time needed to identify it increases exponentially. Even more efficient network motif detection tools have been developed, the time complexity to detect four-nodes motifs in directed graphs is *O(m*^*2*^), where *m* is the number of edges in the network^51^. Furthermore, after obtaining the motifs, it takes time to enumerate all cases for each motif pattern. The enumeration process involving comparing whether two graphs are ‘isomorphic’ is also ‘NP’ hard, and the run-time of the best known algorithm is 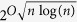 for graphs with *n* vertices^52^. Therefore, it takes much long time to identify larger motifs and enumerate all cases of each motif. What’s more, three-nodes motifs, which can be assembled into four-nodes or larger network motifs, are known as the most basic patterns of regulation with biological meanings^53^. The approach proposed here can also be applied to larger motifs with increasing computational power in the future.

## Materials and Methods

### Data sources and construction of PTRN

Human phosphorylation/dephosphorylation annotations were retrieved from five public resources, i.e. Phospho.ELM (v9.0)^54^, NetworKIN (v2.0)^55^, PhosphoPOINT (downloaded April 2011)^56^, Kinasource (downloaded March 2011) (http://www.kinasource.co.uk) and PhosphoSitePlus (downloaded April 2011)^40^, as well as two systematic studies^3^,^39^. As a result, we obtained 30,258 phosphorylation/dephosphorylation regulations between 5,638 proteins, which encompassed 375 kinases/phosphatases and their 5,601 substrate proteins (Supplementary Table S10). The proteolysis data were extracted from the MEROPS database^57^, which is a major resource that curates proteolytic events. After integrating the data from MEROPS and a previous study^3^, we constructed a proteolytic network composed of 3,672 regulations among 1,920 proteins, including 205 proteases and 1,814 substrates (Supplementary Table S11).

By integrating the above phosphorylation/dephosphorylation and proteolysis regulations, we further constructed a PTRN with each node denotes a protein and an edge links a kinase/phosphatase/protease to its corresponding substrate(s). Considering the possible regulatory interplay between a pair of enzymes, e.g. kinase and kinase/phosphatase, we lay bidirectional edges between such pairs of enzymes while the edges between the rest kinases/phosphatases/proteases and their substrates are unidirectional. Finally, we obtained a PTRN composed of 33,930 regulations among 6,412 proteins, including 375 kinases/phosphatases and 205 proteases.

The drug therapy information and drug-protein interactions were extracted from DrugBank^36^, where the drug therapeutic effects were described with the ATC classification system (ATC codes at the first level were considered).

### Identification of characteristic network motifs

Based on the PTRN constructed above, motifs occurring in the network were identified with FANMOD^9^. Due to the high computational cost of detecting motifs with more nodes from the PTRN, we considered only three-nodes motifs here. To identify the characteristic network motifs, we compared the occurrence frequency (*N*_*real*_) of each three-nodes subnetwork in the PTRN with that in 1,000 randomized networks (*N*_*rand*_), where each edge was rewired while retaining the same node degree distribution when generating the random networks. Each subnetwork was evaluated using two metrics: the *p*-value and *Z*-score. The *p*-value indicates the significance of the subnetwork and the *Z*-score describes the difference between the frequencies of the subnetworks in the real network (*N*_*real*_) and random networks (*N*_*rand*_) as defined below.


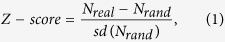


where *sd*(*N*_*rand*_) is the standard deviation of *N*_*rand*_. The subnetworks with *p*-value < 0.05 and *Z*-score >2 were considered to be significant network motifs for further analysis^9^.

### Therapeutic similarity between individual drugs

For drugs that target an interacting protein pair, we assumed that these drugs were therapeutically similar. As shown in Fig. 5, given two proteins targeted by two drugs 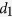 and 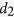, the similarity between the two drugs *T*(*d*_1_,*d*_2_) can be defined as follows.


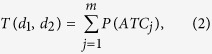



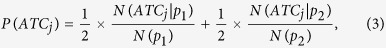


where *ATC*_*j*_ denotes the ATC code *j, d*_1_ and *d*_2_ represent the two drugs that respectively target proteins *p*_1_ and *p*_2_, *m* is the number of the common ATC codes associated with both drugs *d*_1_ and *d*_2_, 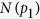 and 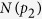 are the numbers of drugs that separately target proteins *p*_1_ and *p*_2_, and 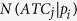 denotes the number of drugs annotated with ATC code *j* targeting protein *i*. 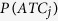 denotes the similarity of drugs *d*_1_ and *d*_2_ with respect to ATC code *j*. The disease gene information was retrieved from the OMIM database^58^.

### Predicting potential drug targets

The network motifs were highly conserved and enriched with drug targets, thus we explored whether it was possible to predict novel drug targets using these motifs. For each motif, we only considered the drugs whose target proteins were significantly enriched in the motif and we predicted the proteins that could be possibly targeted by these drugs. For example, given a new protein in motif I, we compared it with the set of proteins *T* with the same topological structures in motif I. If the function of the new protein is similar to those of proteins from *T*, the protein is likely to interact with drugs targeting *T*, where the functional similarity between a pair of proteins was defined as follows.


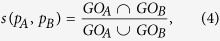


where *A* and *B* are two proteins with the same topological structure in the same motif, and *GO*_*A*_ and *GO*_*B*_ denote the annotations associated with proteins *A* and *B*, respectively. The annotations were obtained from the Gene Ontology (GO) database^59^.

To assess the performance of our approach, we compared it with the popular nearest profile method^37^, which assumes that proteins with high sequence identity will be targeted by the same drug(s)^60^. Here, for fair comparison, we considered functional similarity instead of sequence similarity for the nearest profile approach, which was entitled as NNfun hereinafter. To evaluate the performance of distinct methods for predicting drug targets, we employed the *F*1 score defined as below.


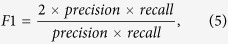


where *precision* is the percentage of predicted positives that are true positives and *recall* is the percentage of true positives that are predicted correctly.

## Additional Information

**How to cite this article**: Zhang, X.-D. *et al.* The exploration of network motifs as potential drug targets from post-translational regulatory networks. *Sci. Rep.* **6**, 20558; doi: 10.1038/srep20558 (2016).

## Supplementary Material

Supplementary Information

Supplementary Table S1

Supplementary Table S2

Supplementary Table S3

Supplementary Table S4

Supplementary Table S5

Supplementary Table S6

Supplementary Table S7

Supplementary Table S8

Supplementary Table S9

Supplementary Table S10

Supplementary Table S11

## Figures and Tables

**Table 1:** Therapeutic categories of drugs that significantly target PTRN motifs.

**Motif**	**Therapeutic categories of drugs targeting motifs (first level of the ATC code)**	**Significance (adjusted** ***p*****-value)**
I	L: Antineoplastic and immunomodulating agents	0.0688
II	L: Antineoplastic and immunomodulating agents	6.550e-4
	A: Alimentary tract and metabolism	0.0023
III	A: Alimentary tract and metabolism	7.390e-4
	L: Antineoplastic and immunomodulating agents	9.020e-4
IV	L: Antineoplastic and immunomodulating agents	3.159e-5
	A: Alimentary tract and metabolism	0.0066
	B: Blood and blood forming organs	0.0142
V	R: Respiratory system	0.0015
	C: Cardiovascular system	0.0186
	L: Antineoplastic and immunomodulating agents	0.0199
	D: Dermatologicals	0.0566
	N: Nervous system	0.0971
VI	L: Antineoplastic and immunomodulating agents	4.423e-4
	A: Alimentary tract and metabolism	0.0856

**Table 2:** Four cases with the same drug that target interacting protein pairs in a parallel manner from motif I.

**Target 1**	**Target 2**	**Third protein**	**Drug 1**	**Drug 2**
FYN	SRC	ADAM15	Dasatinib	Dasatinib
LCK	FYN	ADAM15	Dasatinib	Dasatinib
LCK	SRC	MUC1	Dasatinib	Dasatinib
LCK	SRC	ADAM15	Dasatinib	Dasatinib

**Table 3:** Performance of NetTar, NNfun and Zhao *et al.*’s^38^

Therapeutic category (ATC code)	Data source^a^	NetTar			NNfun			Zhao *et al.*’s^38^		
		***Recall***	***Precision***	***F1***	***Recall***	***Precision***	***F1***	***Recall***	***Precision***	***F1***
A	(1)	0.3636	0.1125	**0.1719**	0.3146	0.0409	0.0724	0.0059	0.0357	0.0101
	(2)	N/A	N/A	N/A	0.3257	0.0441	**0.0777**	0.0065	0.0270	0.0106
	(3)	N/A	N/A	N/A	N/A	N/A	N/A	0.1000	0.0064	**0.0121**
B	(1)	0.4000	0.1538	**0.2222**	0.1681	0.0917	0.1187	0.0860	0.0628	0.0726
	(2)	N/A	N/A	N/A	N/A	N/A	N/A	0.0948	0.0773	**0.0852**
	(3)	0.6833	0.0796	**0.1426**	0.2266	0.0548	0.0883	0.1192	0.1056	0.1120
C	(1)	0.1845	0.0900	0.1210	0.1176	0.1590	**0.1352**	0.0915	0.0010	0.0020
	(2)	0.1789	0.2328	**0.2023**	0.19	0.0379	0.0632	0.0084	0.0009	0.0017
	(3)	0.0952	0.2307	**0.1348**	0.0985	0.0357	0.0524	0.0571	0.0013	0.0026
D	(1)	0.7347	0.0238	**0.0462**	0.3333	0.0147	0.0283	0.0327	0.0022	0.0042
	(2)	N/A	N/A	N/A	N/A	N/A	N/A	0.1064	0.0009	**0.0018**
	(3)	0.5294	0.0654	**0.1165**	0.1578	0.0185	0.0331	0.0416	0.0063	0.0109
L	(1)	0.4109	0.1165	**0.1815**	0.2713	0.0596	0.0978	0.0269	0.0897	0.0414
	(2)	0.3504	0.1108	**0.1683**	0.2436	0.0434	0.0737	0.0182	0.0213	0.0197
	(3)	0.2727	0.2051	**0.2341**	0.2	0.0732	0.1072	0.0288	0.0269	0.0278
N	(1)	0.2935	0.0903	**0.1381**	0.2952	0.0512	0.0873	0.2566	0.0018	0.0036
	(2)	0.3125	0.2403	**0.2717**	0.3048	0.0485	0.0837	0.1996	0.0015	0.0030
	(3)	N/A	N/A	N/A	N/A	N/A	N/A	0.0674	0.0039	**0.0075**
R	(1)	0.1923	0.0327	**0.0558**	0.1379	0.0192	0.0337	0.1681	0.0014	0.0028
	(2)	N/A	N/A	N/A	N/A	N/A	N/A	0.2209	0.0008	**0.0017**
	(3)	0.1052	0.16	**0.1269**	0.0769	0.0247	0.0375	0.1463	0.0030	0.0059
Overall	(1)	0.3692	0.0851	**0.1284**	0.2225	0.0654	0.0833	0.1112	0.0324	0.0227
	(2)	0.2806	0.1936	**0.2141**	0.2660	0.0434	0.0745	0.1091	0.0216	0.0206
	(3)	0.3371	0.1481	**0.1509**	0.1519	0.0413	0.0637	0.0934	0.0255	0.0298

In predicting the target proteins of drugs with distinct therapeutic effects, where N/A means no predictions available. ^a^Data source (1) is the PTRN network, (2) is the network consists of kinase-protein regulations from Tan *et al.*^39^ and protease data from MEROPS database^57^ and Lopez-Otin *et al.*^3^, (3) is the network consists of kinase-protein regulations from PhosphoSitePlus^40^ and protease data from MEROPS database^57^ and Lopez-Otin *et al.*^3^.

**Table 4:** The validation of predicted target proteins by NetTar in public databases.

**Therapeutic category (ATC code)**	**Drug**	**Predicted target proteins**	**Validated source**
A	Vitamin A	CTNNB1; RDH10	STITCH
	Potassium Chloride	NCL; SLC9A8	STITCH
	L-Glutamic Acid	PSMD9; PSMD8; PSMD5; PSMD4; PSMF1; AIMP2; PSMD3; CTPS2; PRL; PSMA2; PSMA3; RARS; PSMA6; PSMA7; PSMA4; PSMA5; PSMC1; PSMC3; PSMC4; PSMD7; NME1; PLCB3; SLC38A1; TPP2; PSME2; PSMB7; UNC13B; CCBL1; PSMD14; SEC61B; CASP3; RIMS1; RASGRF1; PSPH; NPEPPS; PSMD11; PSMD10; PSMD12; BLMH; SYT1; LNPEP; PSMB6; GFPT2; PSMB4; PSMB3; EEF1E1	STITCH
	Papaverine	PDE3B	STITCH
	Pyridoxine	PNPO	STITCH
	Metformin	STK11; EIF4EBP1; IGFBP1	STITCH
	Cisapride	KCNA5	STITCH
	Pioglitazone	EP300; PPARGC1A; NR1H3	STITCH
	Lidocaine	ICAM5	STITCH
	Mesalazine	ALOX15	STITCH
	Sulfasalazine	NFKB2	TTD
B	L-Lysine	TP53	STITCH
C	Isoproterenol	IGF1; TP53; CASP3; FOS; CDKN1B; HRAS; STAT3; NAMPT; MAPK14; UCP1; KRAS; SLC27A1; CD79A	STITCH
		FN1	TTD
	Verapamil	KCNA5	STITCH
	Icosapent	CYP1A1; HMGCS2; APOB; HMGCS1; FABP4; TGS1	STITCH
	Dipyridamole	AHCY	STITCH
	Amrinone	PDE3B	STITCH
	Dopamine	SLC9A3R1; GHRH; NPY; PDYN	STITCH
	Norepinephrine	S1PR1; GNAQ; ARHGEF1	STITCH
	Digoxin	SLCO4A1	STITCH
	Niacin	APOA1	STITCH
	Carvedilol	EDN1	STITCH
	Lidocaine	ICAM5	STITCH
	Bepridil	KCNA5	STITCH
D	Isoproterenol	IGF1; TP53; CASP3; FOS; CDKN1B; HRAS; STAT3; NAMPT; MAPK14; UCP1; KRAS; SLC27A1; CD79A	STITCH
		FN1	TTD
	Ethanol	CS; CYP1A1; ATP5H; LBR; TK1	STITCH
	Tretinoin	HOXC8; PDGFB; BMP4; APBB1IP; ZBTB16; SLC44A1; SKAP2; CDKN1A; RDH10; HOXB7; SMAD2; GRN; TNFRSF10A; PDE6A; CDK4; CYP2C18; S100A11; UGT1A5; AGT; GATA2; PKD1	STITCH
	Isotretinoin	NR1D1; LMNA; SMAD3	STITCH
	Alitretinoin	HOXC8; TRIM16; CDKN1A; SMAD2; SMAD3; IL1A; NR1D1; AGT; NCOR2; NCOR1; NKX2-5; APOB; ZBTB16; RARS; TFRC; PML; DUSP1; NR1H2; NR1H3; HOXB7; TNIP1; LMNA; EP300; GATA2; ID2; NOTCH1; CTGF; CREBBP	STITCH
	Morphine	IL12A	STITCH
	Lidocaine	ICAM5	STITCH
L	Doxorubicin	BRCA1	STITCH
	Imatinib	DDR2; JAK2; KITLG; CLK4; PDGFB	STITCH
	Paclitaxel	BCL2L11; KIF5B; TUBB2A; KIF1A; TUBB2B	STITCH
	Tretinoin	HOXC8; PDGFB; BMP4; APBB1IP; ZBTB16; SLC44A1; SKAP2; CDKN1A; RDH10; HOXB7; SMAD2; GRN; TNFRSF10A; PDE6A; CDK4; CYP2C18; S100A11; UGT1A5; AGT; GATA2; PKD1	STITCH
	Arsenic trioxide	CYP1A1	STITCH
	Alitretinoin	HOXC8; TRIM16; CDKN1A; SMAD2; SMAD3; IL1A; NR1D1; AGT; NCOR2; NCOR1; NKX2-5; APOB; ZBTB16; RARS; TFRC; PML; DUSP1; NR1H2; NR1H3; HOXB7; TNIP1; LMNA; EP300; GATA2; ID2; NOTCH1; CTGF; CREBBP	STITCH
	Celecoxib	BCAR1	STITCH
	Diethylstilbestrol	SFRP2; NCOA3; KLF10; HIC1; PHB; PARP1; ITGB3BP; FASLG; CCK; AGTR2; NCOR2	STITCH
	Estradiol	NCOA3; NR5A1; RUNX2; FASLG; CCK; NCOR2	STITCH
	Sorafenib	STK36; PTK2; CASP3; CRKL; XIAP; MKNK1; PLK4; CASP9; MLTK; MAPK15; DYRK3; TEK; MAP2K5; AURKC; NTRK3; FRS2; BCR	STITCH
	Dasatinib	CASP7; EPHB1; SIK1; EPHB4; SIK2; SYK; EPHA5; TEC; EPHA3; EPHA8; PLCG2; STAT5A; JAK2; PXN; BLK; HCK; STAT3; NEK11; FES; MINK1	STITCH
	Bicalutamide	NKX3-1	STITCH
N	Diazepam	ALB	STITCH
	Pentobarbital	ALB	STITCH
	L-Tryptophan	RPS13; RPS15; PSMD11; PSMD12; PSMA3; RPS3; PSMA7; PSMB7; PSMC1; PSMC5	STITCH
	Choline	SLC44A1; CHAT	STITCH
	Amitriptyline	CRHR1	STITCH
	Nefazodone	CASP7	STITCH
	Caffeine	PPP1R1B	STITCH
	Lidocaine	ICAM5	STITCH
	Lamotrigine	SCN8A	STITCH
R	Morphine	IL12A	STITCH
	L-Cysteine	CNDP2; BCKDHA	STITCH
	Choline	SLC44A1; CHAT	STITCH
	Theophylline	LIPE; GAST	STITCH
	Lidocaine	ICAM5	STITCH
