# Supplementary material for: The exploration of network motifs as potential drug targets from post-translational regulatory networks
Source: Sci Rep. 2016 Feb 8;6:20558. doi: 10.1038/srep20558 (PMC4744934; doi:10.1038/srep20558)
Supplement: Supplementary Information [file srep20558-s1.pdf]

Supplementary information for

The exploration of network motifs as potential drug targets from post-translational regulatory networks

Xiao-Dong Zhang[1,2,†], Jiangning Song[3,4,†], Peer Bork[5,*], and Xing-Ming Zhao[1,*]

[1]Department of Computer Science, School of Electronics and Information Engineering, Tongji University, Shanghai 201804, China

[2]Shanghai Water (Ocean) Administrative Service Center, Shanghai 200050, China

[3]Tianjin Institute of Industrial Biotechnology, Chinese Academy of Sciences, Tianjin 300308, China

[4]Department of Biochemistry and Molecular Biology, Faculty of Medicine, Monash University, Melbourne, VIC 3800, Australia

[5]European Molecular Biology Laboratory, Meyerhofstraße 1, Heidelberg 69117, Germany

*Correspondence and requests for materials should be addressed to X.M.Z. (xm_zhao@tongji.edu.cn) or P.B. (bork@embl.de)

[†]These authors contributed equally to this work.

Supplementary Table S1. Motif I identified with the FANMOD tool. The detailed information about member proteins of motif I.

Supplementary Table S2. Motif II identified with the FANMOD tool. The detailed information about member proteins of motif II.

Supplementary Table S3. Motif III identified with the FANMOD tool. The detailed information about member proteins of motif III.

Supplementary Table S4. Motif IV identified with the FANMOD tool. The detailed information about member proteins of motif IV.

Supplementary Table S5. Motif V identified with the FANMOD tool. The detailed information about member proteins of motif V.

Supplementary Table S6. Motif VI identified with the FANMOD tool. The detailed information about member proteins of motif VI.

Supplementary Table S7. The motif types in which kinases/phosphatases and proteases act as drug targets.

Supplementary Table S8. Cases of six motif that can be used as targets of multi-target agents or combinatorial drugs, where the drugs target the interacting protein pairs tend to share similar therapeutic effects. The first three columns list proteins in motif; the fourth and fifth columns are interacting protein pair targeted by drugs; the sixth is the therapeutic similarity denoted by the first level of ATC code; and the last one lists the common drug(s) (denoted with Drugbank ID) if any of the drugs targeting the protein pair are the same.

Supplementary Table S9. The novel drug targets predicted by NetTar, and the validation of predictions with information from TTD and STITCH database.

Supplementary Table S10. The human phosphorylation regulation network. The first column lists kinases/phosphatases, and the second column lists their corresponding substrates.

Supplementary Table S11. The human proteolysis regulation network. The first column lists proteases, and the second column lists their corresponding substrates.
